# A 3-year follow-up clinical study on the preservation for vitality of involved tooth in jaw cysts through an innovative method

**DOI:** 10.1038/s41598-023-50523-4

**Published:** 2024-01-02

**Authors:** Gang Niu, GongHang Zhang, Jia-min Chen, Tao Wang, Ye Wu, You-Guang Lu, Li-song Lin

**Affiliations:** 1 Department of Maxillofacial Surgery, School and Hospital of Stomatology, Fujian Medical University, 246 Yangqiao Middle Road, Fuzhou, 350001 China; 2 Fujian Key Laboratory of Oral Diseases & Fujian Provincial Engineering Research Center of Oral Biomaterial & Stomatological Key Laboratory of Fujian College and University, School and Hospital of Stomatology, Fujian Medical University, Fuzhou, China; 3 School of Stomatology, Fujian Medical University, Fuzhou, 350004 China; 4 Department of Preventive Dentistry, Fujian Key Laboratory of Oral Diseases, School and Hospital of Stomatology, Fujian Medical University, 246 Yangqiao Middle Road, Fuzhou, 350001 China; 5 Department of Oral and Maxillofacial Surgery, The First Affiliated Hospital of Fujian Medical University, Fuzhou, China

**Keywords:** Nervous system, Oral anatomy, Oral diseases, Oral manifestations

## Abstract

Jaw cysts commonly affect the oral and maxillofacial region, involving adjacent tooth roots. The management of these teeth, particularly regarding root canal therapy and apicoectomy, lacks consensus. This study introduces a novel treatment concept and refined surgical approach to preserve pulp viability in teeth involved in jaw cysts. The objective was to investigate the effectiveness and potential benefits of this approach over a 36-month follow-up period. A conservative management approach prioritized vitality preservation, reserving root canal treatment and apicectomy for cases with post-operative discomfort. A comprehensive follow-up of 108 involved teeth from 36 jaw cyst cases treated with the modified method was conducted. Clinical observation, X-ray imaging, cone-beam computed tomography (CBCT), and pulp vitality testing assessed changes in cyst size, tooth color, pulp vitality, root structure, and surrounding alveolar bone. After 36 months, our modified surgical approach successfully preserved tooth vitality in 84 involved teeth. Adverse symptoms in 19 teeth, such as redness, swelling, fistula, and pain, resolved with postoperative root canal therapy. Follow-up was lost for five teeth in two cases. No cyst recurrences were observed, and in 34 cases, the bone cavity gradually disappeared, restoring normal bone density during long-term follow-up. Our modified surgical method effectively preserves tooth vitality in jaw cysts. This innovative approach has the potential to improve the management of teeth involved in jaw cysts.

## Introduction

Jaw cysts are prevalent conditions within the oral and maxillofacial region^1^. They can be classified into odontogenic cysts, non-odontogenic cysts, and pseudocysts. Odontogenic cysts further encompass inflammatory and developmental subtypes, with radicular cysts being the most frequently encountered inflammatory odontogenic cysts, while dentigerous cysts and odontogenic keratocysts fall under the developmental cyst category^2^. Surgical intervention serves as the primary treatment modality for jaw cysts^3^. In cases of keratocysts, which have a high recurrence rate, meticulous surgical curettage and thorough management of the surrounding bone are recommended^4^. Conversely, for other types of jaw cysts lacking the biological characteristics of keratocysts, preservation of the involved teeth is generally prioritized, except in instances where complete resorption of the surrounding alveolar bone has occurred, warranting extraction^2,5^. Current conventional surgical approaches for jaw cysts involve cyst enucleation, and if the apex of a tooth is exposed within the cyst, root canal therapy and apicoectomy are typically performed^2,5^.

Radicular cysts arise as a result of granulomas or chronic inflammation surrounding the root apex^6,7^. Consequently, these teeth often exhibit a history of caries, trauma, or crown defects, with visible changes in crown color. Radiographic examinations reveal enlarged or resorbed apical foramina, and the pulp is typically necrotic, classifying these teeth as pathogenic. In the management of radicular cysts, preoperative root canal therapy and apicoectomy on the affected teeth have gained wide acceptance within the scholarly community^8^. During the progression of odontogenic jaw cysts, in addition to the pathogenic teeth, the growth and expansion of the cyst can also impact adjacent teeth, resulting in exposure of the root tips within the cystic cavity. These teeth exhibit structurally intact crowns with normal coloration, no radiographic evidence of apical enlargement or absorption, and display either normal or diminished pulp vitality upon testing. Similar findings are observed in non-odontogenic cysts and pseudocysts. Such teeth are referred to as involved teeth.

The involved teeth become encapsulated or compressed during the development of jaw cysts, and the necessity for root canal therapy and apicoectomy remains inconclusive. In clinical practice, it is often observed that fresh blood flow is present in the pulp of involved teeth, despite their pulp vitality tests indicating non-viability. Furthermore, during fenestration decompression of jaw cystic lesions, we have observed the restoration and preservation of tooth vitality and tissue structure in the involved teeth. Preserving tooth vitality not only prolongs the lifespan of the involved teeth but also significantly reduces surgical trauma and treatment costs for patients. Given the advancements in functional oral surgery, the preservation of tooth vitality in involved teeth has emerged as a key research focus in the functional treatment of jaw cysts.

Therefore, it is reasonable to hypothesis that the pulp vitality of involved teeth in jaw cysts can be promisingly to be preserved, contingent upon the application of an appropriate methodology. In this study, we aimed to preserve the vitality of involved tooth in jaw cysts through conducting a thorough and systematic assessment of the involved tooth pulp vitality protected by our modified surgical approach from maxillary cysts over a follow-up period of up to 36 months. In contrast to conventional methods to some degree, our approach involved a modified jaw cyst enucleation method with apicoectomy only in the pathogenic teeth. Specifically, a root canal surgery was conducted solely in the pathogenic teeth before preceding the cyst surgery to facilitate an intraoperative apicoectomy of them. The osteotomy procedure was executed with a 5 mm open window positioned above the root apex to optimize the preservation of involved teeth pulp vitality.

## Results

### Sample profile

A total of 36 patients with jaw cysts, including 21 males and 15 females, were selected for this study. The age of the included patients ranged from 20 to 53 years. Ultimately, 108 involved teeth from these 36 patients met the inclusion and exclusion criteria and were included in the study.

### Pathology diagnosis

**Figure 1:**
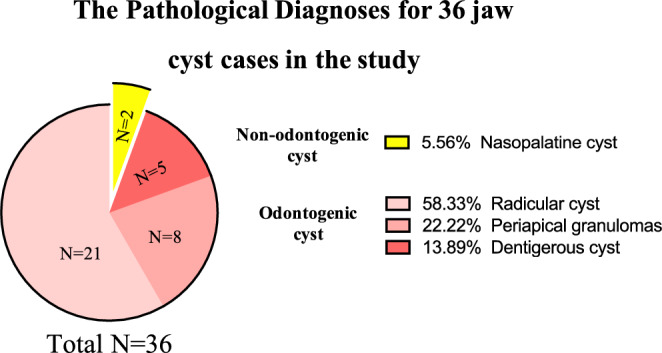
The pathological diagnoses for 36 jaw cyst cases in the study.

**Figure 2:**
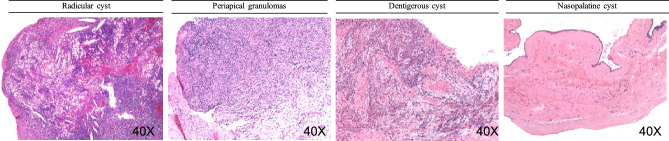
Representative histopathological images showing the characteristics of the jaw cysts.

Based on the pathology diagnosis, the 36 patients in this study were classified as follows: 21 cases of radicular cysts, eight cases of periapical granulomas, five cases of dentigerous cysts, and two cases of nasopalatine cysts. Combining these findings with the patients' clinical manifestations, a final diagnosis of 34 cases of odontogenic cysts and two cases of non-odontogenic cysts was determined (Supplemental Table 1, Figs. 1, 2).

### Pain intensity

**Figure 3:**
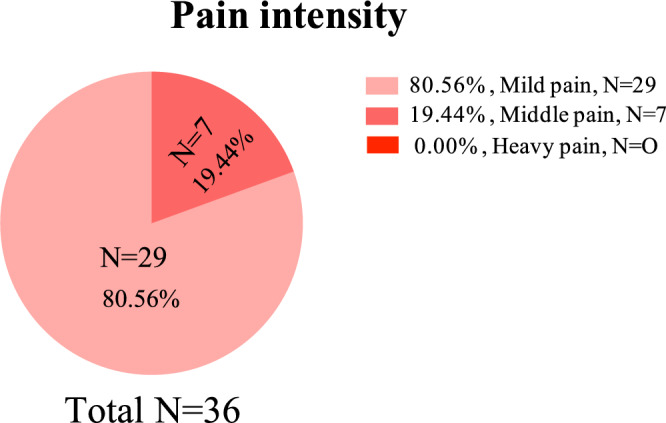
The pain intensity of Visual Analogue Scale (VAS) on the first day after the surgery.

Pain intensity was assessed in all patients on the first day after the surgery using the Visual Analogue Scale (VAS)^6^. The recorded pain levels for each patient were as follows: mild pain (VAS 1–2) was reported by 29 patients (80.56%), moderate pain (VAS 3–4) was reported by 7 patients (19.44%), and no patients reported heavy pain (VAS 5–6) (0%) (Fig. 3).

### Follow-up observation

**Figure 4:**
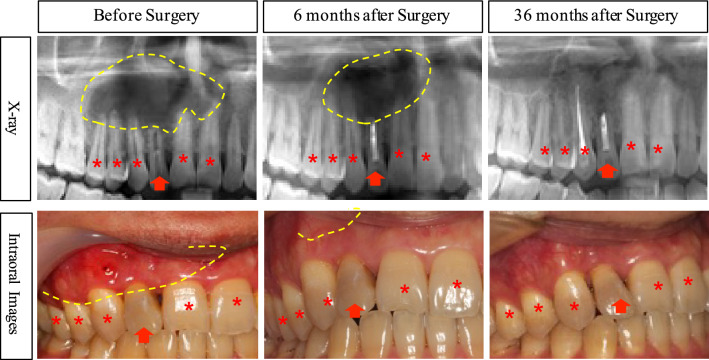
Representative images demonstrating the changes in the jaw cysts and the status of the teeth during the follow-up period. (Red arrows denote focal teeth, red asterisks indicate involved teeth, yellow dotted lines symbolize cyst.).

Two out of the 36 patients, accounting for three involved teeth in one case and two in the others, were lost to follow-up, leaving a total of 34 patients and 103 involved teeth included in the follow-up analysis. Over the course of three years, the surgical wounds gradually healed, and the size of the jaw cyst lesions significantly reduced. No cases of recurrence or postoperative discomfort were observed (Table 1, Fig. 4).

**Table 1:** Observation and record of the postoperative incision healing and the operation results of the jaw cyst.

	Swollen and purulent			Cyst cavity decreased		
	Cases (N)	Percentage (%)	(95% CI)	Cases (N)	Percentage (%)	(95% CI)
Preoperative	9	25.0 (9/36)	0.109, 0.391	0	0.00 (0/36)	0.000, 0.000
Post-1 M	6	17.1 (6/35)	0.047, 0.296	0	0.00 (0/35)	0.000, 0.000
Post-3 Ms	4	11.8 (4/34)	0.009, 0.226	11	32.4 (11/34)	0.166, 0.481
Post-6 Ms	3	8.80 (3/34)	− 0.007, 0.184	23	67.6 (23/34)	0.519, 0.834
Post-12 Ms	0	0.00 (0/34)	0.000, 0.000	28	82.4 (28/34)	0.695, 0.952
Post-24 Ms	0	0.00 (0/34)	0.000, 0.000	34	100 (34/34)	1.000, 1.000
Post-36 Ms	0	0.00 (0/34)	0.000, 0.000	34	100 (34/34)	1.000, 1.000

*M* Month, *Ms* Months, *Post-1 M* 1 month after the surgical operative procedure, *CI* Confidence Interval.

**Figure 5:**
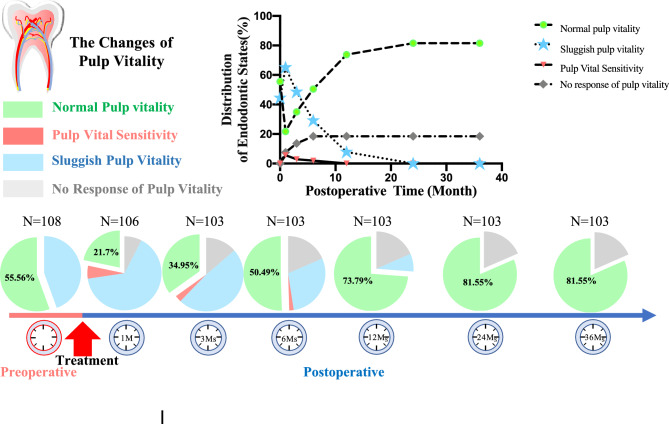
The alterations in the vitality of involved teeth throughout the follow-up period.

Among the 103 involved teeth, 84 of them maintained their vitality after surgery, while 19 teeth eventually lost their vitality. Within one month after the operation, gingival swelling and fistula were observed in eight teeth from six patients. Within three months, gingival swelling and pain were reported in six teeth from four patients. At the 6-month mark, gingival swelling and pain were observed in five teeth from three patients. Despite four weeks of observation, the symptoms in all 19 involved teeth from 13 patients did not subside. Upon clinical examination, X-ray analysis, and pulp vitality testing, these teeth were identified as requiring root canal treatment. After the root canal procedure, the symptoms disappeared within two weeks. The roots of all 103 involved teeth were preserved, with 84 teeth maintaining normal pulp vitality (Table 2, Fig. 5) and no changes in tooth color (Table 3, Fig. 4).

**Table 2:** Observation and record of the involved teeth vitality.

	Normal pulp vitality				Pulp vitality sensitivity			Sluggish pulp vitality			No response of pulp vitality		
	Total (N)	Teeth (n)	n/N (%)	95%CI	Teeth (n)	n/N (%)	95%CI	Teeth (n)	n/N (%)	95%CI	Teeth (n)	n/N (%)	95%CI
Preoperative	108	60	55.6	0.462, 0.649	0	0	0.000, 0.000	48	44.4	0.351, 0.538	0	0.00	0.000, 0.000
Post-1 M	106	23	21.7	0.139, 0.295	6	5.7	0.013, 0.101	69	65.1	0.560, 0.742	8	7.50	0.025, 0.126
Post-3 Ms	103	36	35.0	0.257, 0.442	3	2.9	− 0.003, 0.062	50	48.5	0.389, 0.582	14	13.6	0.070, 0.202
Post-6 Ms	103	52	50.5	0.408, 0.601	2	1.9	− 0.007, 0.046	30	29.1	0.204, 0.379	19	18.4	0.110, 0.259
Post-12 Ms	103	76	73.8	0.653, 0.823	0	0	0.000, 0.000	8	7.80	0.026, 0.129	19	18.4	0.110, 0.259
Post-24 Ms	103	84	81.6	0.741, 0.890	0	0.00	0.000, 0.000	0	0.00	0.000, 0.000	19	18.4	0.110, 0.259
Post-36 Ms	103	84	81.6	0.741, 0.890	0	0.00	0.000, 0.000	0	0.00	0.000, 0.000	19	18.4	0.110, 0.259

*M* Month, *Ms* Months, *Post-1 M* 1 month after the surgical operative procedure.

**Table 3:** The changes of involved teeth crown color.

	Crown surface discoloration (teeth)	Crown surface unchanged (teeth)
Preoperative	0	108
Postoperative (1 month)	0	106
Postoperative (3 months)	0	103
Postoperative (6 months)	0	103
Postoperative (12 months)	0	103
Postoperative (24 months)	5	98
Postoperative (36 months)	19	84

Through additional analysis and statistical examination, no significant difference was observed in the post-surgical prognosis of jaw cysts between cases involving multiple involved teeth and those involving a single tooth (Table 4). Similarly, no statistically significant variance was noted in the pulpal non-responsiveness of the involved teeth (Table 5).

**Table 4:** Comparative prognostic evaluation of jaw cysts involving singular versus multiple involved teeth.

	Jaw cysts singular involved tooth				Jaw cysts singular multiple teeth				_χ_^2^_(P value)_
	Cases (N1)	Cyst cavity decreased (n1)	n1/N1 (%)	95% CI	Cases (N2)	Cyst cavity decreased (n2)	n2/N2 (%)	95% CI	
Post-1 M	4	0	0	0.000, 0.000	31	0	0	0.000, 0.000	–
Post-3 Ms	4	1	25	− 0.174, 0.674	30	10	33.3	0.165, 0.502	0.11199 (0.73789)
Post-6 Ms	4	1	25	− 0.174, 0.674	30	22	73.3	0.575, 0.892	3.76733 (0.05226)
Post-12 Ms	4	2	50	0.010, 0.990	30	26	86.7	0.745, 0.988	3.26508 (0.0777)
Post-24 Ms	4	4	100	1.000, 1.000	30	30	100.0	1.000, 1.000	–
Post-36 Ms	4	4	100	1.000, 1.000	30	30	100.0	1.000, 1.000	–

**Table 5:** Vitality prognostic assessment in maxillary cysts with single vs. multiple involved teeth.

	Jaw cysts singular involved tooth				Jaw cysts singular multiple teeth				_χ_^2^_(P value)_
	Teeth (N1)	No response of pulp vitality (n1)	n1/N1 (%)	95% CI	Teeth (N2)	No response of pulp vitality (n2)	n2/N2 (%)	95% CI	
Post-1 M	4	0	0	0.000, 0.000	106	8	7.50	0.000, 0.000	0.32556 (0.56828)
Post-3 Ms	4	1	25	− 0.037, 0.117	103	13	12.6	0.062, 0.190	0.51882 (0.47135)
Post-6 Ms	4	1	25	− 0.037, 0.117	103	18	17.5	0.101, 0.248	0.14927 (0.69923)
Post-12 Ms	4	1	25	− 0.037, 0.117	103	18	17.5	0.101, 0.248	0.14927 (0.69923)
Post-24 Ms	4	1	25	− 0.037, 0.117	103	18	17.5	0.101, 0.248	0.14927 (0.69923)
Post-36 Ms	4	1	25	− 0.037, 0.117	103	18	17.5	0.101, 0.248	0.14927 (0.69923)

*M* Month, *Ms* Months, *Post-1 M* 1 month after the surgical operative procedure.

## Discussion

In light of our study's findings, we can substantiate the hypothesis that the pulp vitality of involved teeth in jaw cysts can be promisingly to be preserved, contingent upon the implementation of our adapted surgical method.

The objectives of jaw cyst resection are to halt the pathological process, promote the regeneration of jaw bone tissue, and restore the anatomical and physiological functions of the teeth within the cystic area. Inflammatory factors and cystic exudation are known to contribute to the formation of odontogenic cysts^6^, and it is widely accepted that pathogenic tooth root canal treatment and apicoectomy are necessary to prevent postoperative infection and cyst recurrence^7^. According to traditional surgical methods, root canal therapy and apicoectomy are recommended whenever the tooth root is exposed in the cyst cavity, regardless of pulp vitality^3^. While this approach reduces the risk of postoperative infection in the affected area, it also compromises the anatomical structure and vitality of the involved teeth, leading to decreased stability and functional performance^9^. However, in our clinical experience, we have observed fresh blood flow during pulp exposure in the majority of involved teeth. Furthermore, intraoperative observations have revealed no signs of inflammation or adhesion between the root apex and the cystic lesion. Based on these findings, it is possible to properly treat the involved teeth, thereby preserving and restoring their anatomical structure and vitality.

With advancements in oral research and an improved understanding of the significance of tooth vitality, researchers have conducted in-depth investigations on the teeth involved in jaw cysts. Some researchers advocate treating the involved teeth in the same manner as the pathogenic teeth to prevent cyst recurrence^10–12^. Others propose that cyst recurrence is attributed to the microenvironment of the cyst remnants and capsule cavity, which is not directly associated with the involved teeth^13^. Additionally, some scholars have found that in cases of developmental odontogenic jaw cysts, simply resecting the cyst without treating the involved teeth did not result in postoperative infection^14,15^. Therefore, there is still no consensus regarding the treatment of teeth involved in jaw cysts.

In clinical dental practice, the standard for root canal therapy is usually based on pulp sensibility tests (thermal and electric), however, which failed to assess blood flow within in the pulp, but only reflects the conduction function of nerve fibers in the pulp tissue^10^. In light of the foregoing, it is imperative to acknowledge that the present test is susceptible to various interference factors, demonstrates suboptimal accuracy, and manifests elevated incidences of both false positives and false negatives. This is particularly evident in cases involving immature teeth or those that have undergone injuries resulting in the temporary impairment of sensory nerve function^16,17^. Currently, research found that the blood circulation of the tooth was considered a more reliable indicator of pulp vitality than the nervous system^18^. Consequently, alternative tests, including Laser Doppler flowmetry (LDF), pulse oximetry (PO), Dual Wavelength Spectrophotometry (DWLS), and the like, have been incorporated into clinical dental practice, aiming to ascertain a more dependable method for the assessment of pulp vitality. Nevertheless, the intricate nature of the tooth's root canal system, the variable aspects inherent in pulpal vitality, and the substantial cost associated with these emerging techniques have collectively impeded their widespread adoption within clinical practice^16^. Frankly, if we can use these new methods to follow up the involved tooth pulp vitality, it will be benefited a lot to our study.

In terms of the management of teeth associated with cystic lesions of the jaws, there was controversies regarding preservation of the involved teeth with/without root canal treatment^19–22^. Previous papers^23^ pointed that it was essential determination the treatment approach by evaluation of cystic lesions and involved teeth comprehensively (clinical evaluation, image evaluation and pulp vitality testing), which was consistent to the pre-surgical preparation in this study. Liao et al.^24^ evaluated the tooth vitality of 237 involved teeth of 42 patients with odontogenic keratocyst, where root canal therapy was not received for more than 6-months postoperative follow-up. Positive pulp vitality in the study group of marsupialisation in combination with secondary enucleation (71.56%) was significantly higher than that of simple enucleation (36.72%) (*P* < 0.01). The results were quite similar to what we found in this study that 81.55% of the involved teeth preserved vitality and no cyst recurrence. Some researchers also reported complete excision of large peri- apical cyst can be performed without sacrificing the vitality of the adjacent teeth by preserving the integrity of their neurovascular supply through controlled microsurgical enucleation, and by a potential apical vascular repair ensuing unintended injury^25,26^. In one recently reported case, the root canals of included teeth were treated before cyst enucleation, even though the teeth responded to the cold test and the histologic findings confirmed that the extirpated pulps were vital^27^. Nevertheless, due to the devitalised teeth are more vulnerable and prone to being lost later in life, all effort should be made to avoid unnecessary devitalization.

In this study, we implemented several modifications to the surgical methods in order to preserve the anatomical structure and vitality of the involved teeth during the operation. Specifically: ① Prior to surgery, a diagnosis of the involved teeth and pathogenic teeth was made, and root canal therapy was not performed on the involved teeth. ② To expose the cyst during the operation, bone removal was conducted 5 mm above the root apex of the involved tooth to safeguard the nerve and blood supply in the apex, while preserving the integrity and vitality of pulp stem cells and periodontal membrane cells. ③ In the area surrounding the involved teeth, only the epithelial lining of the capsule wall and a portion of the fiber lining were incised under direct vision. Partial retention of the fiber lining was performed to avoid compromising papillary stem cells, multifunctional pulp stem cells, periodontal stem cells, and blood vessels in the root apex. ④ During the operation, complete removal of the cyst and root apex of the pathogenic teeth was carried out, while those of the involved teeth were not excised or cauterized. Irrigation with sterile saline solution was performed to ensure a sterile environment and remove inflammatory factors from the cystic space. ⑤ The root apex of the involved teeth was promptly covered with blood clots during the operation, which facilitated the preservation and restoration of tooth vitality.

It is noteworthy that, notwithstanding the preservation of pulp viability in the overwhelming majority (81.55%) of involved teeth following our adapted maxillary cystectomy, there were 19 teeth resolved with postoperative root canal therapy due to no response after 3-year follow up, the maintaining tooth vitality method proposed in this study should be performed with cautious in reminding patients the complications. The no response of pulp vitality in the involved teeth might attributed to the risks of injuring nerve and blood supply of these teeth during the enucleation procedure with a consequent pulp necrosis^28,29^. Besides that, the recurrence of jaw cysts was still a concern. Zhao et al.^30^ retrospectively analysed the recurrence of 19 keratocystic odontogenic tumours and found the recurrent lesion was involved with the roots of the teeth in three out of six cases where teeth were preserved. Therefore, they concluded that the method of operation for some keratocystic odontogenic lesions should be more aggressive. In this study, there was no recurrence of the jaw cysts observed during the 3-year follow up and we would continuously pay close attention on the prognosis of these clinical cases.

Numerous studies have demonstrated the preservation of involved tooth vitality in the treatment of jaw cystic lesions through fenestration decompression^31,32^. In our clinical practice, we have also observed the flow of fresh blood when opening the involved teeth. It is well known that dental pulp is rich in blood vessels and nerves. Apart from apical vessels, root canal vessels can also communicate with the periodontal ligament and some accessory root canals^33^. During the development of jaw cysts, the involved teeth may experience compression from the cyst and stimulation from inflammatory factors, potentially leading to the presence of Aδ and C nerve fibers in the pulp tissue. However, it remains unclear whether temporary shock, degeneration, or necrosis occurs in the pulp, or if the pulp blood flow in the involved tooth becomes necrotic or remains normal. To date, no reliable basic studies or clinical case reports have provided a definitive answer. Therefore, the current practice of performing root canal therapy and apical excision solely based on the pulp vitality test and the exposure of the tooth root in the cyst lacks a scientific basis^29,34^. Consequently, the preservation of involved tooth vitality in jaw cysts is an important area for further investigation^35^.

Regrettably, in the initial phase of this investigation, constraints related to cost and technical considerations precluded the implementation of retrograde filling in the affected teeth. Retrograde filling is particularly indicated for conditions such as apical pore hypertrophy, gaps between root canal filling and the root canal wall, absence of a root canal, root canal underfilling, and intricate apical anatomy^36,37^. Apical closure facilitated by bioceramic cements not only serves to isolate the root canal system from periodontal tissues and bone cavities post-cyst curettage but also mitigates microleakage following apicectomy, guards against re-infection, and fosters the stimulation of periapical tissue regeneration^38^. In candid acknowledgment, this aspect constitutes a constraint within our study. In subsequent treatments, we plan to implement apical backfilling to enhance therapeutic outcomes. Furthermore, ongoing monitoring and assessment of the effectiveness of these cases will be conducted.

In conclusion, our results demonstrate that the modified surgical method effectively preserves the vitality of involved teeth during the treatment of jaw cysts. But, in the future study, forthcoming endeavors will encompass the enlargement of the sample size, the implementation of extended longitudinal studies with a specific emphasis on odontogenic and non-odontogenic cysts, and a meticulous assessment of both surgical outcomes and the vitality status of the involved teeth. These initiatives are poised to yield more scientifically rigorous and precise experimental findings.

## Methods

From September 2015 to October 2018, patients with jaw cysts were consecutively admitted to a case series examining the concept of maximum preservation the tooth vitality on the jaw cysts area. The study protocol was approved by the Ethics Committee of the Affiliated Stomatological Hospital of Fujian Medical University (approval no. FMUSS-2015-0022). This study was conducted in accordance with the Helsinki Declaration (2013 version) on experimentation including human subjects.

### Treatment program (Fig. 6)

**Figure 6:**
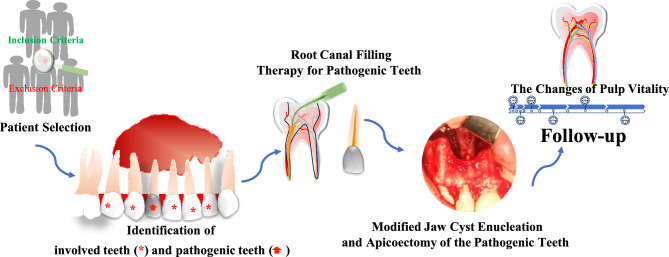
Schematic representation of our treatment program.

The therapeutic procedure proceeded as follows: commencing with a comprehensive assessment based on the patient's complaints, clinical manifestations, and imaging findings, we initially identified the jaw cyst. Subsequently, a meticulous differentiation was undertaken between the involved teeth and the pathogenic teeth. Collaboratively establishing the treatment plan with the patient and their family, informed consent was obtained prior to referring the patient to the endodontics department for the execution of endodontic procedures on the pathogenic tooth.

Following the exclusion of contraindications to surgery, our modified approach involved the enucleation of the jaw cyst, coupled with apicoectomy solely in the pathogenic tooth, performed under local anesthesia or general anesthesia to enhance diagnostic precision. The excised contents of the jaw cyst were submitted to pathology for further diagnostic elucidation. Subsequent observations and follow-ups were conducted to evaluate the prognosis of the cysts and the vitality of the pulp in the involved teeth. In instances where infection and necrosis of the pulp in the involved tooth occurred, root canal treatment was administered, and continued monitoring was implemented to assess the overall prognosis of both the cyst and the affected tooth.

### Patient selection

The subjects were selected in accordance with inclusion and exclusion criteria.

The inclusion criteria were as follows:

① At least 18 years old.
② Cyst diagnoses according to histopathology reports were made following the classification of odontogenic tumors and cysts published by the WHO in 2017^39^.
③ Postoperative pathological diagnosis confirmed the presence of jaw cyst, excluding keratocyst.
④ The root tips of teeth located in the jaw cyst should directly exposed to the cyst.
⑤ Written informed consent was obtained from each patient, and postoperative root canal therapy was provided if any discomfort was reported.

The exclusion criteria were as follows:

① Intraoperative examination revealed that the root tip of teeth was not exposed within the cystic cavity.
② Immediate freeze pathological examination was performed if protein-like substances or yellowish-brown fluid were found in the cyst during surgery. If a diagnosis of keratocyst, ameloblast or glaucoma was confirmed, complete excision was performed immediately.

### Identification of involved teeth and pathogenic

Identification of pathogenic and involved teeth was determined through medical history, clinical examination, and dental radiographs or CBCT scans. Involved teeth should be no enlarged apical foramen, root resorption on X-ray or CBCT (Siemens, Somatom Sensation 16), and demonstrated normal, sluggish or sensitive pulp vitality in pulp vitality tests (Neosono Co-Pilot Pulp Vitality Tester). Contrast to the involved teeth, the pathogenic teeth were observed with peri-apical lesion on both intraoral and radiographical examination. The pathogenic teeth should be treated with complete root canal filling prior to surgery while the involved teeth would not perform the root canal filling therapy. The size, location, and number of involved teeth and pathogenic teeth in the cyst were recorded before surgery.

### Surgical procedure (Figs. 7 and 8)

**Figure 7:**
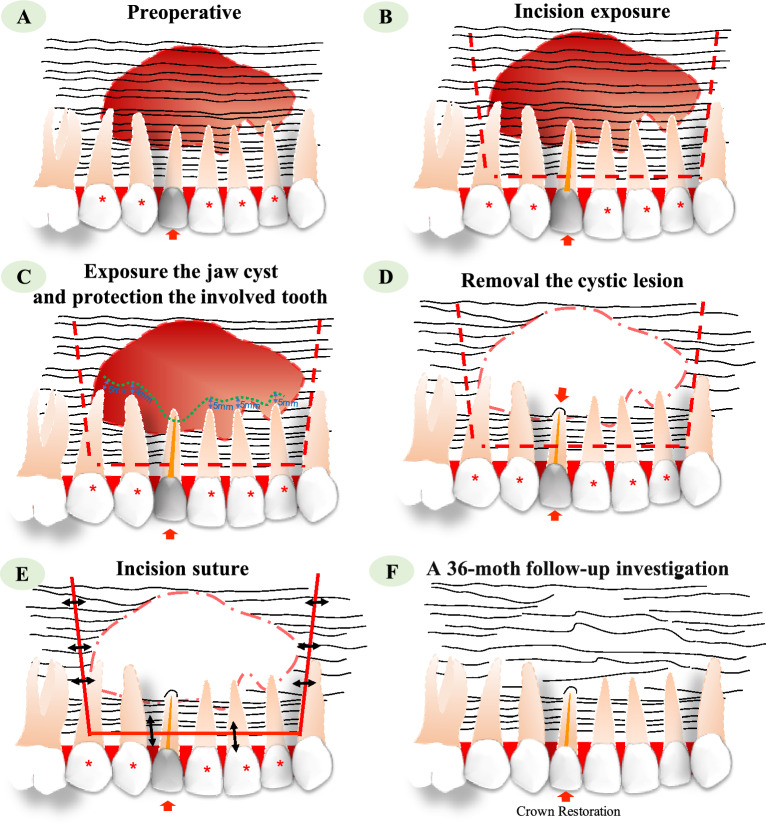
A schematic representation of our modified surgical method employed in this study for the treatment of jaw cysts with exposed teeth (red arrows denote focal teeth, red asterisks indicate involved teeth, red dotted lines symbolize incision sites, and black double arrows represent suture placements). Notably, if the root of the involved tooth was not exposed within the odontogenic cyst cavity or for the non-odontogenic cysts (except for keratocyst, ameloblast and glaucoma), complete cyst removal during surgery was deemed adequate.

**Figure 8:**
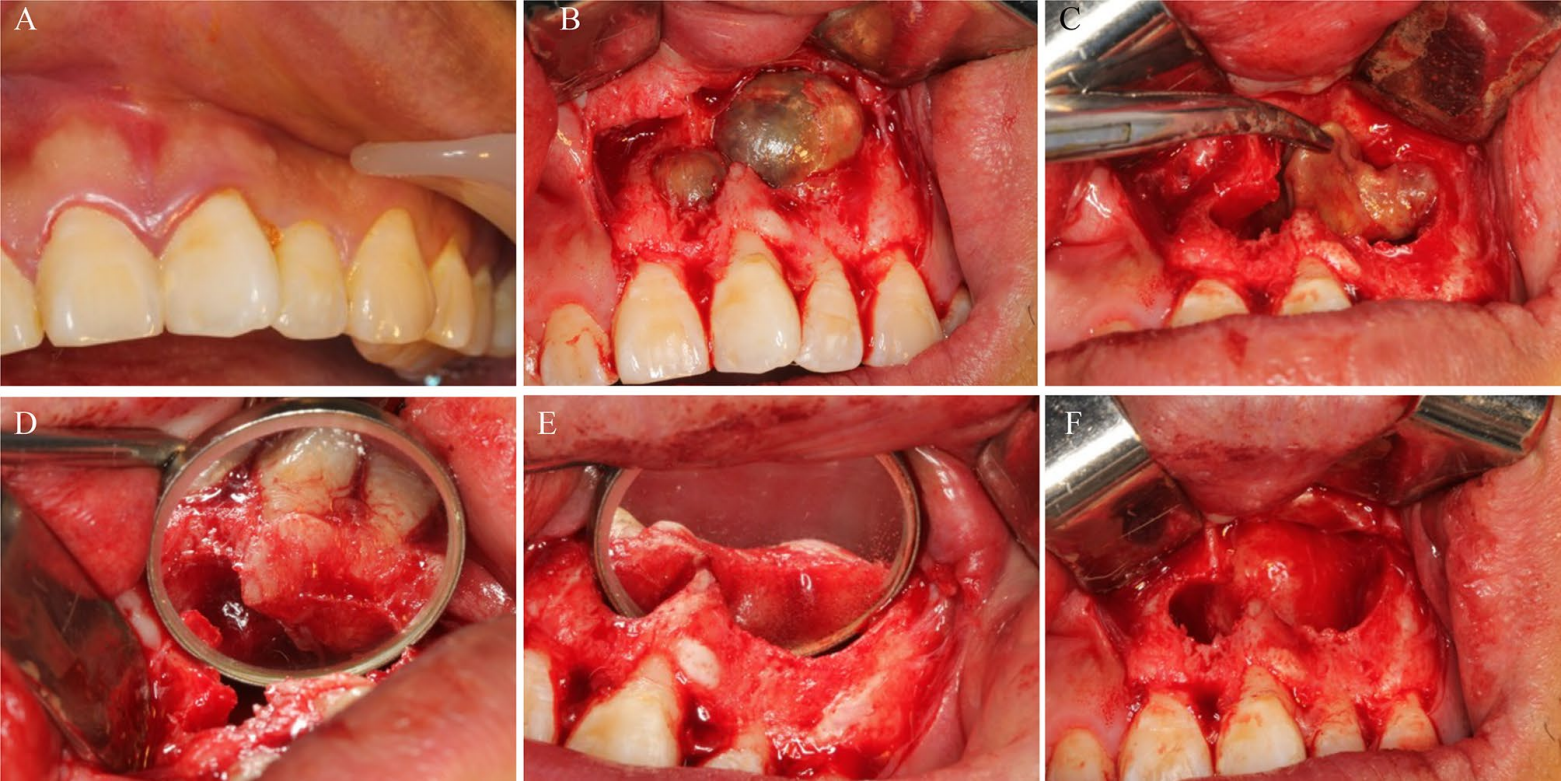
Representative images illustrating the different steps of the surgical procedure. (**A**) Preoperative condition; (**B**) Incision exposure; (**C**) Exposure of the jaw cyst wall; (**D**) Preservation of the tissue around the involved teeth; (**E**) Protection of the root apex and surrounding tissues of the involved teeth.

The surgical procedures were conducted under (Figs. 7 and 8) was performed under whether local anesthesia (4% articaine solution) or general anesthesia (1.5–2.5 mg/kg propofol), depending on the assessment of anesthetist. With the primary goal to maximum preserve the root tip blood of the involved teeth while exposing the cyst, the surgical window was positioned 5 mm above the apex of the involved tooth. As recognized, maintaining a healthy blood circulation is imperative for the preservation of pulp vitality^9,36^. Furthermore, this approach incorporates various measures to mitigate damage to the involved tooth during the surgical procedure (Figs. 7C and 8B).

For the involved teeth, only the epithelial lining of the capsule wall and a portion of the fiber parietal layer were removed with meticulously and under direct vision. It was noteworthy that complete removal of the fiber parietal layer was not recommended since the blood supply and nerve fibers of the involved teeth might be damaged (Fig. 8C,D).

For the pathogenic teeth, a thorough treatment of the area surrounding the pathogenic teeth was conducted. The sac wall attached to the root of the pathogenic tooth, which is difficult to remove, was burned and scraped using an electric knife. Subsequently, the root was resected by 3 mm using round burs, while ensuring the protection of the involved teeth (Fig. 8E). The resected root was covered with a gelatin sponge. If the cavity size was less than three tooth positions, it was sutured after saline irrigation. For cavities larger than four tooth positions, iodoform gauze was placed to fill the cavity before suturing the wound.

### Postoperative evaluation

**Figure 9:**
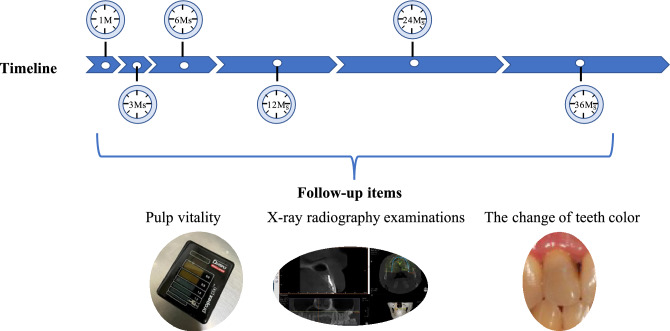
Flowchart illustrating the follow-up procedures and the assessed parameters.

Postoperative evaluation was conducted over a period of 36 months. Follow-up assessments were performed at 1 month, 3 months, 6 months, 12 months, 24 months, and 36 months postoperatively. These assessments included pulp vitality tests, X-ray radiography examinations, and evaluation of tooth color (Fig. 9). The criteria for determining the successful preservation of involved tooth vitality were based on previous reports and are outlined as follows:

- Adequate healing of the surgical incision without any signs of inflammation in the surgical area.
- Proper functioning of the involved teeth in occlusion without any discomfort.
- Restoration of normal pulp vitality in the involved teeth following the operation.
- No observable change in the color of the involved teeth.
- Gradual reduction in the size of the lesion and an increase in bone density.

Failure to meet any of the following criteria within 3 years after surgery indicated unsuccessful preservation of involved tooth vitality:

- Patient experiences toothache or discomfort, requiring root canal therapy.
- Redness, swelling, and pus formation in the surgical area mucosa. After root canal therapy, the inflammatory reaction subsides.
- Lack of reduction or enlargement of the cystic space around the involved teeth as observed in X-ray imaging.

### Statistical analysis

Statistical computations were conducted using SPSS 22.0 software (SPSS Inc., Chicago, IL). Summary statistics were presented as percentages. Ration comparisons were performed using the chi-square test where applicable. Confidence intervals at the 95% level were computed using standard formulae, relying on the asymptotic normality of maximum likelihood estimators and employing the delta method for the logit function. A significance level of p < 0.05 was employed to determine statistical significance.

### Ethics approval and consent to participate

This study was conducted with the approval of the Ethics Committee of the Affiliated Stomatological Hospital of Fujian Medical University (approval no. FMUSS-2015-0022). Written informed consent was obtained from each participant. This study was conducted in accordance with the Helsinki Declaration (2013 version) on experimentation including human subjects.

### Supplementary Information

Supplementary Table 1.

## Data Availability

The datasets generated and/or analyzed during the current study are available from the corresponding author upon reasonable request.
