# Supplementary material for: A 3-year follow-up clinical study on the preservation for vitality of involved tooth in jaw cysts through an innovative method
Source: Sci Rep. 2024 Jan 2;14:128. doi: 10.1038/s41598-023-50523-4 (PMC10761841; doi:10.1038/s41598-023-50523-4)
Supplement: Supplementary file 1 — Supplementary Table 1. [file 41598_2023_50523_MOESM1_ESM.docx]

**Supplemental Table 1: Pathology diagnosis of jaw cysts in this study.**

| **Pathology diagnosis** | | **Number of cases** |
| --- | --- | --- |
| **Odontogenic cyst**  **Non-odontogenic cyst** | Radicular cyst | 21 |
|  | Periapical granulomas | 8 |
|  | Dentigerous cyst | 5 |
| **Non-odontogenic cyst** | Nasopalatine cyst | 2 |
